# Engineering Tissues without the Use of a Synthetic Scaffold: A Twenty-Year History of the Self-Assembly Method

**DOI:** 10.1155/2018/5684679

**Published:** 2018-03-08

**Authors:** Ingrid Saba, Weronika Jakubowska, Stéphane Bolduc, Stéphane Chabaud

**Affiliations:** ^1^Centre de Recherche en Organogenèse Expérimentale de l'Université Laval/LOEX, Centre Hospitalier Universitaire (CHU) de Québec-Université Laval, Hôpital Enfant-Jésus, Québec City, QC, Canada; ^2^Department of Surgery, Faculty of Medicine, Université Laval, Québec City, QC, Canada

## Abstract

Twenty years ago, Dr. François A. Auger, the founder of the Laboratory of Experimental Organogenesis (LOEX), introduced the self-assembly technique. This innovative technique relies on the ability of dermal fibroblasts to produce and assemble their own extracellular matrix, differing from all other tissue-engineering techniques that use preformed synthetic scaffolds. Nevertheless, the use of the self-assembly technique was limited for a long time due to its main drawbacks: time and cost. Recent scientific breakthroughs have addressed these limitations. New protocol modifications that aim at increasing the rate of extracellular matrix formation have been proposed to reduce the production costs and laboratory handling time of engineered tissues. Moreover, the introduction of vascularization strategies* in vitro* permits the formation of capillary-like networks within reconstructed tissues. These optimization strategies enable the large-scale production of inexpensive native-like substitutes using the self-assembly technique. These substitutes can be used to reconstruct three-dimensional models free of exogenous materials for clinical and fundamental applications.

## 1. Introduction

### 1.1. A Brief History of Tissue-Engineering Techniques

Throughout history, as the lifestyle of hunters and gatherers shifted towards a sedentary one, population demographics changed drastically. These changes in lifestyle combined with rich diets lead to an increased lifespan and the emergence of new diseases. The ageing population correlates with an increase in the prevalence of chronic disorders, which represent a large burden on the healthcare system. Chronic disorders can ultimately result in organ failure and may require organ replacement. However, the waiting lists for organ transplantation keep growing every year and the supply of organs does not meet the existing demand. To circumvent this extensive organ shortage, many efforts are being devoted to the reconstruction of tissue-engineered organs [1].

Tissue engineering represents an interesting alternative that aims at reconstructing biological substitutes for the replacement of damaged tissues and organs. This emerging discipline comprises several techniques, most of which use biomaterials such as preformed scaffolds that mimic the morphology of specific tissues and serve as an anchorage point for cells. These scaffolds consist of synthetic or biological materials [2]. Since the biomaterials used for scaffolds are different from the original components of the extracellular matrix (ECM) of the target organs, the differentiation of cells, stem cells in particular, is often partial or inadequate. Also, the use of biomaterials may trigger unknown side effects upon implantation in the human body.

New techniques have recently been developed to improve the functionality of biomaterials, such as the modification of their surface compounds [3], the introduction of bioprinting using bioink made of cells directly embedded in elements of the ECM [4–7], the use of decellularized tissue [8], and, finally, the reconstruction of autologous tissues using only patients' cells.

### 1.2. The Self-Assembly Technique

**Figure 1:**
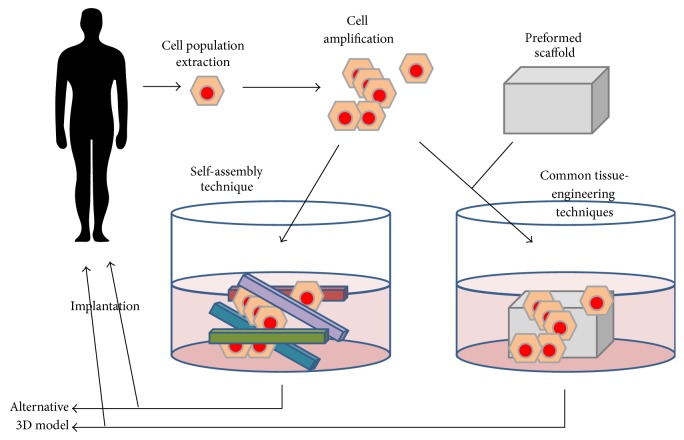
Tissue-engineering strategies. The tissue-engineering strategies are mainly based on cell population extraction from patient's biopsies followed by cell amplification with phenotype maintenance or adequate differentiation. Cells are then seeded on preformed scaffolds, made of synthetic or biologic (including decellularized tissue) biomaterials, and cultivated until grafting. Alternatively, mesenchymal cells can be plated on culture dishes and cultivated in the presence of ascorbate to form their own stroma which could be seeded with epithelial cells. The reconstructed tissue could also serve as accurate models for the study of a wide panel of pathologies.

**Figure 2:**
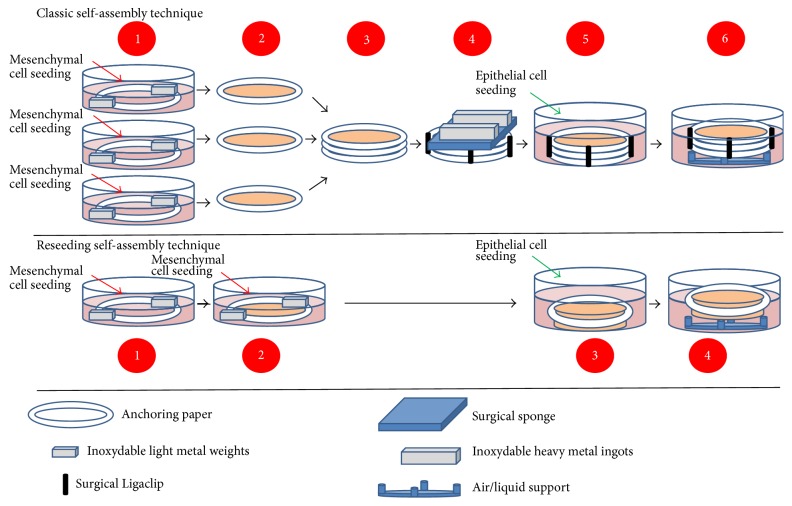
Schema for the production of the “flat” model by the self-assembly technique. Upper panel: classic self-assembly technique. (1) Mesenchymal cells are seeded into 3 cell culture dishes and cultivated for 28 days in the presence of ascorbate. (2) The 3 stromal sheets are peeled from the dish and (3) they are stacked to form a thicker tissue. (4) To ensure fusion of the 3 sheets, a mechanical load is applied on the stacked sheets for a variable time. (5) Then, epithelial cells are seeded on the top of the construct and culture continued for 7 days before (6) tissues are placed at the air/liquid interface for 21 days in order to obtain a mature epithelium. Middle panel: reseeding self-assembly technique. Mesenchymal cells are seeded into cell culture dishes and cultivated for 14 days in the presence of ascorbate before (2) a second seeding of mesenchymal cells on the top of the first stromal sheet. After another 14 days, (3) epithelial cells are seeded on the top of the construct and culture continued for 7 days before (6) tissues are placed at the air/liquid interface for 21 days in order to obtain a mature epithelium. Lower panel: tools required to produce tissue by the self-assembly technique. The anchoring paper is used to limit the contraction of the tissue by the mesenchymal cells and to help in manipulation. The metal weights help to stabilize the anchoring paper during the different cell culture steps. The surgical Ligaclips eliminate the movement of stromal sheets during the fusion step. The surgical sponge is needed to avoid the direct contact between the heavy metal weights and the tissue, and these weights are responsible for the mechanical load favoring the stroma sheet fusion. Finally the air/liquid support maintains the epithelium in contact with the air and the stroma in the cell culture medium.

The self-assembly technique is a method used to reconstruct tissues free of exogenous materials using only patients' cells (Figure 1). The self-assembly technique relies on the ability of mesenchymal cells to secrete and assemble their own ECM. This approach originates from two main discoveries. Firstly, in 1972 ascorbate, an enzymatic cofactor of lysyl- and prolyl-hydroxylase, was shown to stimulate the production of type I collagen by human dermal fibroblasts [9]. Secondly, a key experiment followed in 1989 that demonstrated that fibroblasts can deposit enough ECM within few days to create a three-dimensional (3D) stromal sheet [10]. The stromal sheets can then be stacked or rolled in order to create a 3D organ substitute [11–13]. In the process of tissue reconstruction, cells are extracted from a patient's biopsy and the stroma and the epithelium are enzymatically separated using thermolysin. Mesenchymal cells are extracted from the stroma using collagenase, while epithelial cells are extracted from the epithelium using trypsin. Mesenchymal cells are supplemented with 50 *μ*g/ml of ascorbic acid throughout the cell culture time to enhance the formation of stromal sheets [11]. These newly formed stromal sheets can be easily peeled from the culture dish and stacked to create a cellularized scaffold. The assembled stroma is kept in culture for an additional week to allow further cell-matrix reorganization and layer fusion. Then, epithelial cells are seeded on the top of the construct and the culture is continued for an additional week in order to sustain epithelial cell proliferation and to obtain a full epithelial coverage of the apical surface. Thereafter, the equivalent is maintained for 21 days at an air-liquid interface to induce the maturation of the epithelium [12, 14] (Figure 2). Many different tissues have been produced using this technique such as skin, blood vessel, heart valve, cornea, adipose tissue, urologic tissue, and vaginal mucosa.

#### 1.2.1. Skin

Reconstructed human skin substitutes produced by the self-assembly technique are clinically used for wound healing and burn treatments [15, 16]. The use of reconstructed autologous skin substitutes prevents graft rejection by the immune system. These engineered skin substitutes present a near-to-native architecture and show an adequately differentiated epidermis that is histologically and functionally similar to human skin [12, 17, 18]. Dermal fibroblasts and keratinocytes proliferate* in vitro* as some progenitor stem cells remain present amongst these cell populations [19].

In addition to the clinical potential of the skin substitutes reconstructed by the self-assembly technique, several other fundamental applications have been developed. Engineered substitutes of diseased skin have been produced using cells derived from patients with psoriasis [20]. These constructs can serve as a model for the study of the disease. Accordingly, reconstructed skin made using cells from patients with psoriasis has shown excessive growth and the aberrant differentiation of keratinocytes.

Since the cells used to reconstruct a tissue can be from either a healthy or a diseased donor, the effect of the different cell populations can be investigated individually to assess their effects on disease evolution [20, 21]. Furthermore, the skin substitutes produced with the self-assembly approach have been used to characterize cellular and molecular events involved in the pathogenesis of fibrotic diseases such as hypertrophic scars and scleroderma [22–24]. Also, in the early stage of amyotrophic lateral sclerosis (ALS), patients present with skin alterations. Reconstructed skin substitutes made using the biopsies of ALS patients have been used to identify disease-specific biomarkers which could serve as early diagnostic tools to monitor disease progression [25]. Hence, engineered skin substitutes represent a good alternative to the use of animal models for drug testing and disease modeling [20, 21, 26–28].

#### 1.2.2. Blood Vessels

**Figure 3:**
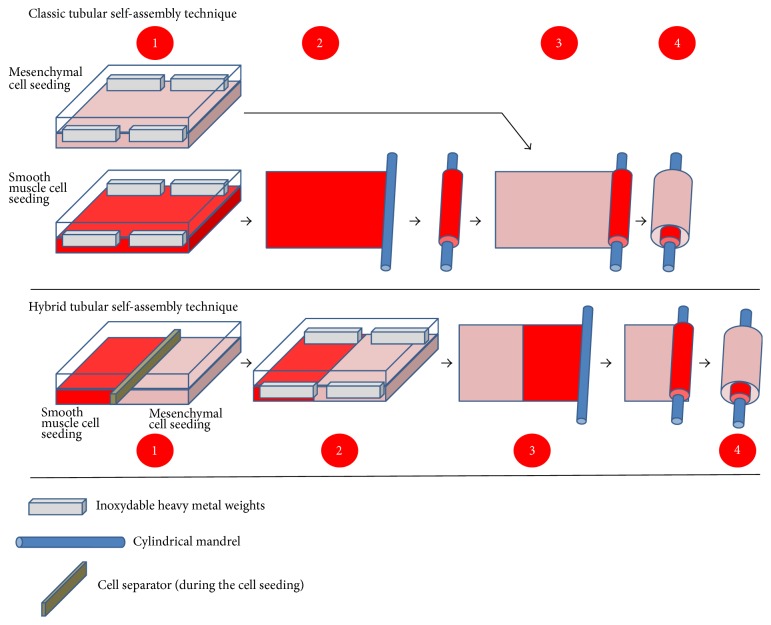
Schema of the production of the “tubular” model by the self-assembly technique. Upper panel: classic tubular self-assembly technique. (1) Mesenchymal cells are seeded in a cell culture plate coated with gelatin. They are cultivated for 28 days in the presence of ascorbate. One plate is seeded with smooth muscle cells (SMC) (in the case of blood vessels) and another with dermal fibroblasts. (2) Then the stromal sheet formed by the SMC is tightly rolled around a cylindrical mandrel followed by (3) the stromal sheet formed by the dermal fibroblasts. (4) The resulting construct is cultivated for a variable time in order to ensure fusion of the rolled sheets. When fusion has been achieved, the mandrel can be removed and the tubular structure could be perfused to be seeded with endothelial cells and allowed to mature. Middle panel: Hybrid tubular self-assembly technique. (1) Instead of using two cell culture plates, each one seeded with a different type of cell population, the plate is separated in two halves with a cell separator. Then each compartment is seeded with one type of cell population: SMC in one part and dermal fibroblasts in the other. (2) After cell adhesion, the separator is removed and cells are cultivated for 28 days in the presence of ascorbate. (3) The stromal sheet is then tightly rolled around the cylindrical mandrel. (4) The resulting construct is cultivated for a variable time in order to ensure fusion of the rolled sheets. When fusion has been achieved, the mandrel can be removed and the tubular structure could be perfused to be seeded with endothelial cells and allowed to mature. Lower panel: tools required to produce tissue by the tubular self-assembly technique. Heavy weights are used to block contraction of the stromal sheets by mesenchymal cells, especially SMC. A cylindrical mandrel is used to create the lumen of the blood vessel. A cell separator divides the cell culture plate in order to create two cellular compartments.

The self-assembly technique has also been applied to engineered human blood vessels [11, 29]. In order to reproduce the tubular shape of blood vessels, the flat stromal sheets produced by the self-assembly technique have been rolled around a cylindrical mandrel. To imitate the physiological layers of blood vessels, a stromal sheet composed of smooth muscle cells was rolled first, followed by a sheet produced by dermal fibroblasts [11, 30, 31] (Figure 3).

#### 1.2.3. Heart Valves

Cardiovascular diseases represent an important problematic in developed countries and their treatment may necessitate heart valve replacement surgery. Rather than replacing a defective or diseased heart valve with a mechanical, cadaver, or animal valve, a lot of effort is being invested in the reconstruction of autologous tissue-engineered heart valves. Although this remains challenging work, heart valves produced using the self-assembly technique have been designed. These stentless bioprosthetic valves could offer a new alternative to artificial valves [32, 33].

#### 1.2.4. Ocular Tissues

Corneal tissue engineering was developed in an attempt to cure corneal opacity. The self-assembly technique has also been applied to the generation of corneal substitutes [34]. These reconstructed substitutes have near-to-native stromal, endothelial, and epithelial histology. The differentiated epithelial layer has well defined basal and wing cells that expressed Na^+^/K^+^ ATPase *α*1 protein, keratin 3/12, and basic keratins [34]. The pattern of MMP gene expression involved in corneal wound healing has also been studied using this model [35]. Also, in an attempt to reconstruct intraocular lenses for the management of ocular diseases, recent studies have emerged that used epithelial cells derived from mouse lenses to generate 3D lentoids in culture [36, 37]. These engineered hollow spheroids produced using gelatin microbeads mimic the 3D microstructure and illustrate the possibility of engineering 3D organoids [36, 37].

#### 1.2.5. Adipose Tissues

Adipose tissues can be produced with the self-assembly technique using adipose-derived stem/stromal cells (ASCs). Cell cultures supplemented with ascorbic acid and adipogenic differentiation factors generate substitutes that share many features with native adipose tissue [38].

#### 1.2.6. Urologic Tissues

The reconstruction of bladder substitutes using the self-assembly technique has been inspired by the skin model, whereas ureter and urethra reconstruction has been based on tubular blood vessel models. Initially, bladder substitutes were produced using a porcine biopsy [39]. The bladder model was later enhanced by combining human dermal fibroblasts with porcine urothelial cells [40]. Substitutes were matured in a bioreactor in order to mimic the filling and voiding cycles of the bladder [41]. As human bladder biopsies became more easily available, similar techniques were used to produce human cell-derived substitutes [42, 43]. Furthermore, urethral substitutes were produced by seeding urothelial cells inside a tubular structure made of dermal stromal sheets [44]. To mimic the* in vivo* tissue architecture, the engineered constructs were perfused in a bioreactor [45]. Moreover, endothelial cells (EC) were also added to these models to reconstruct prevascularized models [46, 47]. These reconstructed urological tissues demonstrate histological and molecular similarities to native tissue. For example, the urothelium expresses uroplakins and zonula-occludens-1 in the epithelial superficial layer. These surface proteins are essential for tissue function as they act as a barrier to prevent urine leakage. These reconstructed urologic substitutes can also serve as invaluable tools for disease modeling. For instance, the bladder model has been used to study the effect of ketamine, a drug that is excreted in the urine. Ketamine was originally used as a medication for anesthesia and chronic pain management. However, the use of ketamine as a recreational drug can cause urinary tract side effects such as cystitis and urinary tract dysfunction [48]. The exposure of the 3D bladder model to ketamine has produced urothelium damage due to induced cell apoptosis [49].

#### 1.2.7. Vaginal Mucosa

The tissue engineering of autologous vaginal mucosa opens the door to new surgical applications for vaginal reconstruction in paediatric patients with congenital urogenital abnormalities such as Müllerian agenesis, also known as the Mayer-Rokitansky-Küster-Hauser syndrome. A new human vaginal mucosa model using the self-assembly technique was recently introduced. This model is derived from human vaginal fibroblasts and epithelial cells, which were extracted from the vaginal biopsies of healthy donors [50]. Cell cultures were supplemented with 17-*β* estradiol in order to reproduce a hormone-responsive setting. The reconstructed tissues showed histological features that were similar to near-native tissue including a fully differentiated stratified squamous vaginal epithelium. Furthermore, vaginal epithelial cells expressed specific markers such as estrogen-*β* receptor and mucin-1. Also, the intermediate epithelial cell layers were filled with polysaccharides such as glycogen. The glycogen content of vaginal epithelial cells is metabolized by lactobacilli, a dominant bacteria of the normal vaginal microbiota that secretes lactic acid.

#### 1.2.8. Bone

The self-assembly technique has recently been used to produce a thin bone substitute from ASCs that were differentiated into osteoblasts. In these tissues, alkaline phosphatase was homogeneously distributed and the substitutes showed a very low contractile capacity. Osteocalcin and hydroxyapatite were found in higher levels within the reconstructed bone from ASC than in non-osteogenically induced tissues [51].

## 2. Improving Stromal Thickness and the Mechanical Properties of Reconstructed Tissues

Although the self-assembly approach is suitable for clinical applications, the time and expenditure required for tissue reconstruction limit its wider use. To circumvent these major drawbacks, new strategies, aimed at increasing the ECM deposition rate and reorganization, have been proposed. In addition to reducing time and costs, these strategies also increase stromal thickness and improve mechanical properties.

### 2.1. Mechanical Stimulation

Major biological modifications in the organization of the cell cytoskeleton and the ECM composition can be induced by mechanical stimuli [52, 53]. For example, blood flow induces the realignment of collagen fibers, which, in turn, strengthens the tissue [54, 55]. These changes are induced by the activation of mechanoreceptors such as the ones containing the arginyl-glycyl-aspartic acid (RGD) integrin binding sites [56]. The signaling pathways of the extracellular signal-regulated kinase, ERK, and the c-Jun N-terminal kinase, c-JNK, are often activated to regulate cellular adaptation to these new environmental conditions [56]. In some studies, G-proteins also seem to be involved in molecular signaling [57]. Cellular responses to mechanical stimuli include the secretion of growth factors such as transforming growth factor-beta (TGF-*β*) [58], which is known to enhance ECM production and reduce matrix metalloproteinase (MMP) secretion and activation. Nevertheless, latent MMPs and other proteases can also be secreted or activated following mechanical stimuli [59, 60]. Fine-tuning is required to tightly control the balance between the synthesis of ECM elements and their degradation. As fibrosis has been observed in a mechanical overstimulation setting, the rate of collagen deposition needs to be controlled to prevent irreversible changes and fibrosis [61].

#### 2.1.1. Cell Cultures in Dynamic Conditions

An appropriate laminar/cyclic flow in a bioreactor improves the quality of tubular constructs as demonstrated in the urethra [44] and blood vessel substitutes [62]. In order to evaluate the effect of dynamic culture conditions on tissue thickness, reconstructed connective substitutes generated with dermal fibroblasts or ASCs were subjected to static or dynamic conditions using a 3D shaker platform (gyrotwister) [63]. Dynamic culture conditions caused a 1.5- to 2-fold increase in the tissue thickness of the substitutes derived from ASCs when compared to static conditions. However, these results did not apply to stromal tissues made of dermal fibroblasts. Hence, it is not possible to conclude that dynamic conditions favor mechanical properties, as they have not yet been extensively measured.

#### 2.1.2. Fiber Alignment within Engineered Tissues

Tissue function can be improved by using microstructured surfaces that control the interactions between cells and the ECM. The surface topography of an elastomeric material can cause a change in the orientation of fibers within multiple cell layers. Here the cell culture surface not only oriented the first cell layer, but also influenced the second cell layer. The latter took on a physiological alignment mimicking the native tissue. As the secreted ECM fibers align according to the orientation of the cells, well-structured stromal sheets can be obtained for cornea, vascular structures, and dermis [64]. Recently, a protocol adapted from the self-assembly technique allowed an easier production of tissues with horizontally aligned collagen fibers, which could be useful for cancer and ageing studies [65].

### 2.2. Enzymatic Reactions and Chemical Stimulation

ECM production can be increased by the supplementation of chemical inhibitors of MMP in cell cultures. The addition of galardin (GM-6001) during the production of skin substitutes significantly increased the thickness of reconstructed tissues [23]. Nevertheless, the use of galardin in the self-assembly technique is restricted because of its high price and its potential impact on the ECM. Galardin acts on the MMPs and could potentially modify the organization of the ECM and, consequently, affect the outcome of the reconstructed tissues.

Furthermore, proline is an important amino acid that is metabolized during collagen synthesis. Its metabolic precursor L-arginine (L-Arg) is converted into ornithine followed by glutamine semialdehyde and finally proline. An L-Arg enriched diet gave significant results in the treatment of wounds* in vivo *[66]. Hence, the effect of L-Arg supplementation on stroma formation was investigated. Although a 20% increase in type I collagen synthesis and secretion was observed, collagen deposition remained unchanged when compared to controls [47]. A plausible explanation for these results could be that the enzymes involved in collagen maturation were not sufficient to process the surplus of this protein* in vitro*.

### 2.3. Biological Stimulation

Biological stimulation of the ECM deposition is a challenge due to the pleiotropic roles of the usable bioactive agents and their subtle effects, which could have a long-term outcome (e.g., after tissue implantation). While experiments using monolayer culture rarely exceed days, studies using the self-assembly technique can last for months, especially if tissues are implanted* in vivo*. Many proteins, peptides, and lipids that are used to stimulate collagen synthesis and deposition must be carefully used to avoid the induction of a pathological situation such as fibrosis or cancer.

#### 2.3.1. Polysaccharides

Beta-glucans, a family of carbohydrates, induce collagen production by fibroblasts [67, 68]. The supplementation of dermal fibroblast cultures with laminaran, which is derived from* Saccharina longicruris *seaweed, increases collagen secretion [69]. Tissues produced using the self-assembly technique with the addition of laminaran showed an increase in collagen synthesis and secretion without inducing a fibrotic phenotype. This results in a thicker stroma without a significant increase in cell proliferation nor in the alpha-smooth muscle actin content [70]. The mechanisms underlying the effect of laminaran could be due to its aggregation properties which could trigger a net increase in collagen secretion [70, 71].

Advanced glycation end-products (AGE) are derived from glucose metabolism and are found within the tissues of elderly or diabetic patients [72]. In cell culture, glucose is the primary source of energy for mesenchymal cells and provides the energy required to sustain ECM production. However, the glucose concentration used in the self-assembly protocol is 4.5-fold higher than normal glycaemia. Consequently, AGE can form within the produced tissues. Also, AGE are involved in the process of skin ageing and have an impact on mechanical and biological parameters [73]. New approaches that circumvent this issue, using glucose-free medium, are currently being developed and should generate promising alternatives [Chabaud et al. unpublished data].

#### 2.3.2. Insulin

Insulin has been used for a long time to safely control the glycaemia of diabetic patients. In addition to its mediating role in glucose entry in cells, insulin also plays an active role in collagen synthesis and deposition [74]. Collagen fiber density in diabetic mouse models can be increased by the use of hydrogels containing microencapsulated insulin-secreting cells [75]. The implantation of PLGA alginate structures that release insulin in rats has also been shown to increase collagen deposition and maturation [76]. Furthermore, wound healing represents a clinical issue for diabetic patients, due in part to the alteration of their insulin metabolism.

#### 2.3.3. Hypoxia

Diabetes is associated with tissue damage such as hypoxia and vascular disease. Evidence suggests that diabetes causes EC loss and that the tissues surrounding capillaries undergo hypoxia. Damaged EC release factors which may induce fibrosis [77]; however other unknown mechanisms also contribute to this change in cell phenotype [74, 78]. Insulin and hypoxia exert a synergic effect on tissues produced by the self-assembly technique. They increase collagen deposition in human and animal cell cultures [79] [Chabaud et al. unpublished data]. Nevertheless, the long-term effect of hypoxia exposure (more than 2 weeks) induced significant acidification of the cell culture medium, even when buffered solutions were used, and resulted in a decrease in tissue thickness [80]. Hence, cyclic hypoxia seems to be a better alternative than constitutive hypoxia as it produces thicker engineered tissues.

#### 2.3.4. Adenosine

Adenosine and other derivatives have been used to enhance the rate of wound healing [81]. Their receptors were also found to be involved in fibrosis. An increase in collagen synthesis and a decrease in MMP-9 activity were observed when A_2B_-adenosine receptors (A_2A_AR) were activated [82, 83]. The production of rabbit tissues by the self-assembly approach in the presence of adenosine resulted in a net collagen content increase and in stromal thickening [79]. The effects of adenosine on human cell cultures remain to be evaluated.

#### 2.3.5. Lysophosphatidic Acid

Lysophosphatidic acid (LPA) is a bioactive lipid present in human blood. LPA expression has been found to be upregulated in fibrosis and cancer [84, 85]. LPA binds to its receptors at the surface of many cells and activates pathways leading to proliferation, migration, and the secretion of cytokines. The culture of dermal fibroblasts with LPA resulted in a dose-dependent increase in type I collagen and fibronectin deposition that could be completely reversed. The addition of LPA to cell cultures did not have any adverse effects on the reconstructed substitutes nor did it influence cell proliferation [42]. The addition of LPA to cell cultures could reduce culture time by up to 25% as it enhances the rate of collagen deposition [42].

#### 2.3.6. The Potential of Adult Stem Cells for Self-Assembly Tissue Engineering

Virtually all primary cell populations contain stem cells or at least potential progenitors [86]. For example, ASC can increase the amount of deposited ECM and, consequently, increase tissue thickness [63]. These cells can also secrete many proangiogenic and immunomodulatory factors [87]. The addition of a stromal sheet made of ASC to the bilayered reconstructed skin could possibly benefit in graft take and subsequent patient management.

Skin reconstruction could also benefit from another kind of adult stem cell, the sox10+ cells. Sox10^+^ adult stem cells are found in the stroma of subcutaneous loose connective tissues. They can differentiate into fibroblasts and myofibroblasts. Myofibroblasts secrete transitory ECM and are eliminated by apoptosis during the resolution phase of wound healing. However, these cells could induce fibrosis of the dermis, resulting in hypertrophic scarring characterized by excessive deposition of the ECM by persistent myofibroblasts [88]. A coculture of endothelial cells and Sox10^+^ stem cells resulted in the differentiation of Sox10^+^ stem cells into perivascular cells that stabilized the newly formed capillary-like network [89]. Although the use of Sox10^+^ stem cells requires a tight control of cell differentiation, they can be beneficial for ECM deposition and for capillary-like structure stabilization.

## 3. Other Improvements of Tissues Produced by the Self-Assembly Technique

### 3.1. Reseeding Self-Assembly Technique

In the original self-assembly technique protocols, several stromal sheets are stacked together to generate a construct with adequate mechanical strength. The mesenchymal cells are seeded on a cell culture compatible surface such as plastic or a gelatin-coated plastic. After cell confluence is reached, fibroblasts begin to deposit ECM. As cells are attached to the plastic surface of the bottom of the wells, the ECM is deposited above them. Thus the cells remain confined at the bottom of the wells and are only partially embedded in the ECM.

**Figure 4:**
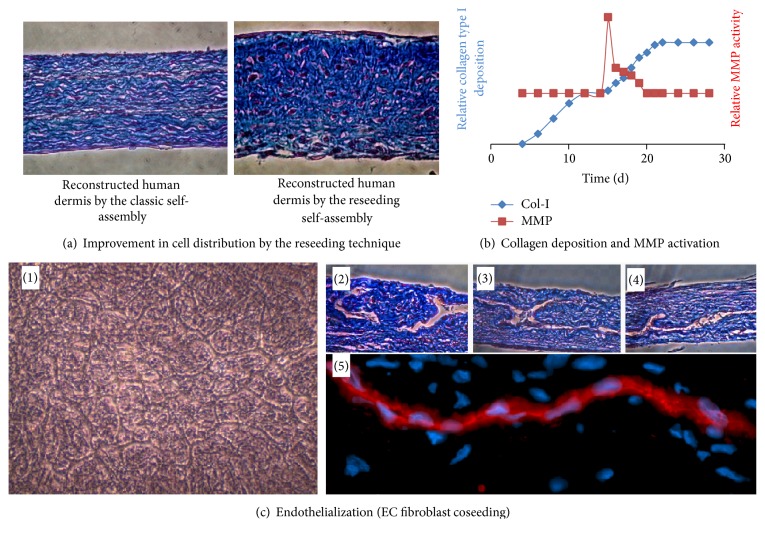
Reseeding self-assembly technique. (a) Photographs of slices of tissue reconstructed by the classic self-assembly technique (left) or the reseeding self-assembly technique (right), stained with Masson's trichrome. Cells are in red/purple and ECM in blue. (b) Graph summarizing the balance between ECM production and its degradation during the production of a stroma by the self-assembly reseeding technique. Collagen deposition was measured by immunoblotting extracts from tissues produced until the indicated time. MMP activity was measured using tissue culture medium harvested at the indicated time using a fluorometric test. (c) (1) Phase contrast photograph of a coculture of endothelial cells and dermal fibroblasts (14 days after seeding). (2), (3), and (4) Photographs of slices of tissue reconstructed by the reseeding self-assembly technique, stained with Masson's trichrome. Three-dimensional capillary-like structures are clearly visible. (5) Photograph of a slice of tissue produced by the reseeding self-assembly technique immunostained using antibodies raised against CD-31.

Although the steps of sheet stacking and fusion induce ECM remodelling, alternating layers of cells and ECM are visible at the sites of sheet stacking across the constructs. The addition of epithelial cells reduces the demarcation between sheets; however it remains visible in 3D tissues. These visible cell layers do not reflect native stroma architecture and could cause delamination [43, 47, 63, 90]. Changes in protocol (Figure 2) were proposed to ensure a more homogenous distribution of cells throughout the tissue and prevent delamination [47] (Figure 4(a)).

#### 3.1.1. The Rationale of Reseeding

When fibroblasts reach confluence, they begin to secrete and deposit collagen to form the ECM. Collagen synthesis reaches a plateau after two weeks because of the inability of fibroblasts to physically assemble collagen fibers [47]. This plateau in the collagen deposition rate also correlates with the achievement of maximal tissue thickness [63]. Hence the idea is that the reseeding of mesenchymal cells on a quiescent 2-week-old stromal sheet would allow the restoration of the rate of collagen deposition and result in the production of thicker tissues.

#### 3.1.2. Reseeding Self-Assembly Approach

**Figure 5:**
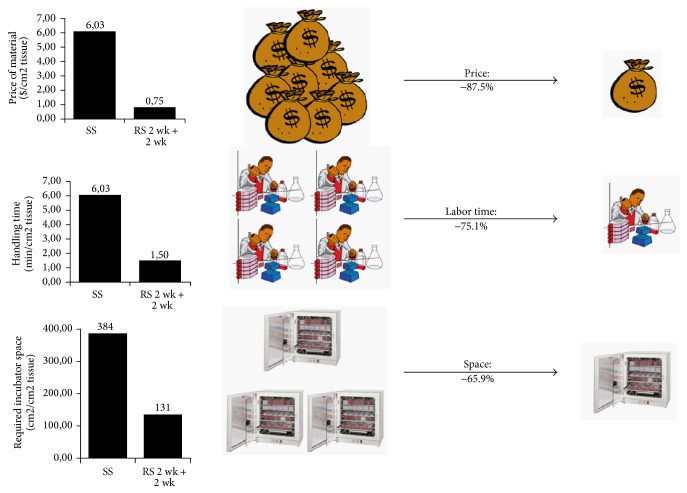
Advantages of the reseeding self-assembly technique as compared to the classic technique. The reseeding self-assembly technique allows a reduction in the costs of the reconstructed stroma by 87.5%, a reduction of the labor time needed for technicians by 75.1%, and a reduction of the volume occupied inside cell culture incubators of 65.9%.

The second layer of fibroblasts seeded onto the first sheet concomitantly induces a transitory peak of MMP activity and a boost in collagen deposition (Figure 4(b)). The fibroblasts within the first sheet layer play a role in the ECM remodelling process. Decellularization of this initial fibroblast layer prior to reseeding has shown a decrease in tissue thickness. The transitory peak of MMP activity observed after the reseeding step could partially contribute to the migration of fibroblasts from the plastic surface, where they were initially seeded, to the interior of the tissue and explain the better distribution of cells amongst the tissues generated using reseeding. After two additional weeks of culture, the single stroma sheets that had undergone reseeding had the same thickness as those that were produced by the sheet stacking method (Figure 4(a)). Furthermore, the reseeded tissues supported the maturation of a fully differentiated epithelium [47]. The reseeding technique offers an alternative to the classical self-assembly protocol which is easier to set up and reduces the costs associated with extensive culture medium consumption [47] as well as required incubator space (Figure 5). This new protocol paves the way to the automation process of the production of tissues by the self-assembly technique as it is expected to reduce costs and generate improved substitutes with optimized quality consistency.

### 3.2. Improvement of Blood Vessel Production

To improve the production of tissue-engineered blood vessels, fibroblasts and SMC were separately cultured in the same flask. Each cell population was grown in its respective half of the flask to generate a construct that was half stroma and half muscle. The sheet was later rolled around a cylindrical stent in such a way that the muscle half formed the interior of the vessel, while the other stroma half formed the external layers of the blood vessel construct (Figure 3). This strategy ensures better fusion between the layers and generates vascular substitutes that have a high-grade mechanical strength to sustain compatibility with engraftment [91–93].

### 3.3. Organ-Specific Stroma

As the interaction between different cell populations is important for tissue homeostasis, the origins of these cells greatly influence mesenchymal-epithelial interactions. The self-assembly technique depends on the ability of mesenchymal cells to secrete the ECM that serves as a scaffold for epithelial cells. Organ-specific cells, which correspond to mesenchymal and epithelial cells derived from the same organ, exhibit specific cell behavior that affects the quality of reconstructed tissues. For example, reconstructed human cornea substitutes showed macroscopic and histological differences depending on the source of mesenchymal cells used: dermal fibroblasts or corneal keratocytes, which are a mesenchymal cell population specific to the cornea [94]. Engineered tissues made with dermal fibroblasts were less transparent and lacked ultraviolet-absorption capability as compared to cornea produced using keratocytes [94]. If mesenchymal cells are important for adequate epithelial differentiation, epithelial cell origin is also important for obtaining an appropriate stroma organization [35].

The use of organ-specific mesenchymal cells instead of dermal fibroblasts to produce bladder mucosa substitutes ensures a better urothelial maturation as there is no expression of cytokeratin-14, a marker absent in native urothelium but present in epidermis, and which causes a significant decrease in ERK phosphorylation, a marker of cell proliferation [95].

The study of organ-specific models can also be applied in the context of disease modeling to identify the effect of the mesenchymal cells on epithelium differentiation. This has been elegantly illustrated with the creation of hybrid psoriasis models using a combination of healthy or psoriatic dermal fibroblasts with healthy or psoriatic keratinocytes. The combination of a stroma derived from psoriatic cells with healthy epithelial cells resulted in an altered epidermis, whereas the combination of a stroma derived from healthy cells with psoriatic epithelial cells resulted in an attenuated epithelial phenotype [20]. Hence, this model has given new insights into the partial effect of mesenchymal cells in the molecular processes involved in psoriasis [20].

## 4. Tissue Engineering with Serum-Free Medium

To this day, most laboratories still use animal serum for cell culture. Although the risks of using animal products have been long discussed, recent emerging pathologies point out that species barriers to the transmission of diseases are not fully supported by scientific arguments. This debate raised concerns about the use of animal products, including serum, to generate implantable devices or organs [96]. The use of animal serum in tissue-engineered organs produced by the self-assembly technique represents a critical issue that impedes clinical translation.

Serum use can impair cell differentiation and cause discrepancies in tissue quality from lot to lot [97, 98]. Human dermal equivalents have been produced by the self-assembly technique using a serum-free medium [99–103]. The resulting tissues presented a similar thickness or even a better one compared to those produced using fetal calf serum, and its organization was characterized at the molecular level. The horizontal development of epidermis was also demonstrated in the absence of serum [104].

## 5. Vascular and Lymphatic Endothelialization of Substitutes Reconstructed by the Self-Assembly Technique

### 5.1. The Endothelialization of Tissues Produced with the Self-Assembly Technique Accelerates Graft Perfusion

The compromised vascularization of reconstructed substitutes upon implantation represents an important cause of graft failure. To prevent graft necrosis, prevascularization strategies were proposed for the reconstructed tissues prior to implantation. The addition of EC to cell culture is a promising solution as EC have the ability to assemble and form capillary-like networks* in vitro. *Human endothelialized reconstructed skin constructs were generated by adapting the self-assembly technique. After four weeks of culture, EC were seeded on the surface of two stromal sheets. Later, these endothelialized stromal sheets were stacked together with an additional nonendothelialized stromal sheet placed at the apical surface of the new construct [105]. The human endothelialized reconstructed skin showed early signs of vascularization after implantation in mice as compared to the nonendothelialized controls. Vascularization in the endothelialized human skin constructs occurred within four days following tissue implantation, whereas it took 14 days in the controls [106]. The colocalization of human and host mouse EC inside a human capillary within the graft suggested the formation of chimeric microvessels and confirmed inosculation between both microvascular networks. Similar results were obtained after the implantation of endothelialized tubular urethral substitutes in mice [46].

### 5.2. The Improvement of Capillary-Like Network Distribution and Maturation

Although the technique previously described for the generation of endothelialized constructs allows the formation of a capillary-like network within the reconstructed tissues, EC remain confined between the stacked cell sheets and form a two-dimensional (2D) vascular network rather than a 3D one. In order to obtain an optimal 3D capillary network, EC were coseeded with dermal fibroblasts [47] (Figure 4(c)). The incorporation of EC in the reconstructed dermis also resulted in an increase in tissue elasticity and mechanical strength. As fibroblasts were seeded at high density, the ECM was quickly generated and rapidly embedded EC inside the stroma [47]. This new endothelialized stroma showed pericyte-like cells surrounding the capillary-like structures that expressed a neuron-glial 2 (NG2) marker.

### 5.3. Formation of a Lymphatic Microvascular Network

In recent studies, a lymphatic capillary network was formed within a dermal construct using the classic self-assembly approach [107, 108]. The integration of such a network can be important in order to rapidly restore lymphatic function within the graft.

## 6. Conclusion

The self-assembly approach has been used to generate several tissues for fundamental and clinical research applications. Over the years, substantial concomitant mechanical and biological improvements of the bioengineered substitutes have been achieved with protocol modifications. For instance, the use of organ-specific mesenchymal cells to produce stroma is associated with better epithelial differentiation. Despite the challenges regarding production costs and time, many strategies have been proposed in order to increase the rate of ECM production and decrease culture time. Finally, the endothelialization of engineered tissues represents a major optimization that could significantly contribute to graft survival.

## References

[1] Langer R., Vacanti J. P. (1993). Tissue engineering. *Science*.

[2] Chan B. P., Leong K. W. (2008). Scaffolding in tissue engineering: general approaches and tissue-specific considerations. *European Spine Journal*.

[3] Minardi S., Taraballi F., Pandolfi L., Tasciotti E. (2016). Patterning Biomaterials for the Spatiotemporal Delivery of Bioactive Molecules. *Frontiers in Bioengineering and Biotechnology*.

[4] Cui H., Nowicki M., Fisher J. P., Zhang L. G. (2017). 3D Bioprinting for Organ Regeneration. *Advanced Healthcare Materials*.

[5] Ji S., Guvendiren M. (2017). Recent Advances in Bioink Design for 3D Bioprinting of Tissues and Organs. *Frontiers in Bioengineering and Biotechnology*.

[6] Li J., Chen M., Fan X., Zhou H. (2016). Recent advances in bioprinting techniques: Approaches, applications and future prospects. *Journal of Translational Medicine*.

[7] Wang X., Ao Q., Tian X. (2016). 3D bioprinting technologies for hard tissue and organ engineering. *Materials *.

[8] Gilpin A., Yang Y. (2017). Decellularization Strategies for Regenerative Medicine: From Processing Techniques to Applications. *BioMed Research International*.

[9] Switzer B. R., Summer G. K. (1972). Collagen synthesis in human skin fibroblasts: effects of ascorbate, -ketoglutarate and ferrous ion on proline hydroxylation.. *Journal of Nutrition*.

[10] Hata R.-I., Senoo H. (1989). L-ascorbic acid 2-phosphate stimulates collagen accumulation, cell proliferation, and formation of a three-dimensional tissuelike substance by skin fibroblasts. *Journal of Cellular Physiology*.

[11] L'Heureux N., Pâquet S., Labbé R., Germain L., Auger F. A. (1998). A completely biological tissue-engineered human blood vessel. *The FASEB Journal*.

[12] Michel M., L'Heureux N., Pouliot R., Xu W., Auger F. A., Germain L. (1999). Characterization of a new tissue-engineered human skin equivalent with hair. *In Vitro Cellular & Developmental Biology - Animal*.

[13] Germain L., Auger F. A., Grandbois E. (1999). Reconstructed human cornea produced in vitro by tissue engineering. *Pathobiology*.

[14] Pruniéras M., Régnier M., Woodley D. (1983). Methods for cultivation of keratinocytes with an air-liquid interface. *Journal of Investigative Dermatology*.

[15] Boa O., Cloutier C. B., Genest H. (2013). Prospective study on the treatment of lower-extremity chronic venous and mixed ulcers using tissue-engineered skin substitute made by the self-assembly approach. *Advances in Skin & Wound Care*.

[16] Beaudoin Cloutier C., Guignard R., Bernard G. (2015). Production of a Bilayered Self-Assembled Skin Substitute Using a Tissue-Engineered Acellular Dermal Matrix. *Tissue Engineering - Part C: Methods*.

[17] Cvetkovska B., Islam N., Goulet F., Germain L. (2009). Identification of functional markers in a self-assembled skin substitute in vitro. *In Vitro Cellular & Developmental Biology - Animal*.

[18] Pouliot R., Larouche D., Auger F. A. (2002). Reconstructed human skin produced in vitro and grafted on athymic mice. *Transplantation*.

[19] Lavoie A., Fugère C., Beauparlant A. (2013). Human epithelial stem cells persist within tissue-engineered skin produced by the self-assembly approach. *Tissue Engineering Part: A*.

[20] Jean J., Lapointe M., Soucy J., Pouliot R. (2009). Development of an in vitro psoriatic skin model by tissue engineering. *Journal of Dermatological Science*.

[21] Jean J., Leroy M., Duque-Fernandez A., Bernard G., Soucy J., Pouliot R. (2015). Characterization of a psoriatic skin model produced with involved or uninvolved cells. *Journal of Tissue Engineering and Regenerative Medicine*.

[22] Bellemare J., Roberge C. J., Bergeron D., Lopez-Vallé C. A., Roy M., Moulin V. J. (2005). Epidermis promotes dermal fibrosis: Role in the pathogenesis of hypertrophic scars. *The Journal of Pathology*.

[23] Simon F., Bergeron D., Larochelle S. (2012). Enhanced secretion of TIMP-1 by human hypertrophic scar keratinocytes could contribute to fibrosis. *Burns*.

[24] Corriveau M.-P., Boufaied I., Lessard J. (2009). The fibrotic phenotype of systemic sclerosis fibroblasts varies with disease duration and severity of skin involvement: reconstitution of skin fibrosis development using a tissue engineering approach. *The Journal of Pathology*.

[25] Paré B., Touzel-Deschênes L., Lamontagne R. (2015). Early detection of structural abnormalities and cytoplasmic accumulation of TDP-43 in tissue-engineered skins derived from ALS patients. *Acta Neuropathologica Communications*.

[26] Jean J., Soucy J., Pouliot R. (2011). Effects of retinoic acid on keratinocyte proliferation and differentiation in a psoriatic skin model. *Tissue Engineering Part A*.

[27] Ayata R. E., Bouhout S., Auger M., Pouliot R. (2014). Study of in vitro capillary-like structures in psoriatic skin substitutes. *BioResearch Open Access*.

[28] García-Pérez M.-E., Royer M., Duque-Fernandez A., Diouf P. N., Stevanovic T., Pouliot R. (2010). Antioxidant, toxicological and antiproliferative properties of Canadian polyphenolic extracts on normal and psoriatic keratinocytes. *Journal of Ethnopharmacology*.

[29] L'Heureux N., Germain L., Labbé R., Auger F. A. (1993). In vitro construction of a human blood vessel from cultured vascular cells: A morphologic study. *Journal of Vascular Surgery*.

[30] Grenier G., Rémy-Zolghadri M., Guignard R. (2003). Isolation and culture of the three vascular cell types from a small vein biopsy sample. *In Vitro Cellular & Developmental Biology - Animal*.

[31] L'Heureux N., Stoclet J.-C., Auger F. A., Lagaud G. J.-L., Germain L., Andriantsitohaina R. (2001). A human tissue-engineered vascular media: a new model for pharmacological studies of contractile responses. *The FASEB Journal*.

[32] Tremblay C., Ruel J., Bourget J.-M. (2014). A new construction technique for tissue-engineered heart valves using the self-Assembly method. *Tissue Engineering - Part C: Methods*.

[33] Dubé J., Bourget J.-M., Gauvin R. (2014). Progress in developing a living human tissue-engineered tri-leaflet heart valve assembled from tissue produced by the self-assembly approach. *Acta Biomaterialia*.

[34] Proulx S., d'Arc Uwamaliya J., Carrier P. (2010). Reconstruction of a human cornea by the self-assembly approach of tissue engineering using the three native cell types. *Molecular Vision*.

[35] Couture C., Zaniolo K., Carrier P. (2016). The tissue-engineered human cornea as a model to study expression of matrix metalloproteinases during corneal wound healing. *Biomaterials*.

[36] Wang D., Wang E., Liu K., Xia C.-H., Li S., Gong X. (2017). Roles of TGF*β* and FGF signals during growth and differentiation of mouse lens epithelial cell in vitro. *Scientific Reports*.

[37] Wang E., Wang D., Geng A., Seo R., Gong X. (2017). Growth of hollow cell spheroids in microbead templated chambers. *Biomaterials*.

[38] Vermette M., Trottier V., Ménard V., Saint-Pierre L., Roy A., Fradette J. (2007). Production of a new tissue-engineered adipose substitute from human adipose-derived stromal cells. *Biomaterials*.

[39] Magnan M., Berthod F., Champigny M.-F., Soucy F., Bolduc S. (2006). In vitro reconstruction of a tissue-engineered endothelialized bladder from a single porcine biopsy. *Journal of Pediatric Urology*.

[40] Bouhout S., Perron E., Gauvin R. (2010). In vitro reconstruction of an autologous, watertight, and resistant vesical equivalent. *Tissue Engineering Part: A*.

[41] Bouhout S., Gauvin R., Gibot L., Aubé D., Bolduc S. (2011). Bladder substitute reconstructed in a physiological pressure environment. *Journal of Pediatric Urology*.

[42] Chabaud S., Marcoux T. L., Deschênes-Rompré M. P. (2015). Lysophosphatidic acid enhances collagen deposition and matrix thickening in engineered tissue. *Journal of Tissue Engineering and Regenerative Medicine*.

[43] Rousseau A., Fradette J., Bernard G., Gauvin R., Laterreur V., Bolduc S. (2013). Adipose-derived stromal cells for the reconstruction of a human vesical equivalent. *Journal of Tissue Engineering and Regenerative Medicine*.

[44] Magnan M., Lévesque P., Gauvin R. (2009). Tissue engineering of a genitourinary tubular tissue graft resistant to suturing and high internal pressures. *Tissue Engineering Part: A*.

[45] Cattan V., Bernard G., Rousseau A. (2011). Mechanical stimuli-induced urothelial differentiation in a human tissue-engineered tubular genitourinary graft. *European Urology*.

[46] Imbeault A., Bernard G., Rousseau A. (2013). An endothelialized urothelial cell-seeded tubular graft for urethral replacement. *Canadian Urological Association Journal*.

[47] Chabaud S., Rousseau A., Marcoux T., Bolduc S. (2015). Inexpensive production of near-native engineered stromas. *Journal of Tissue Engineering and Regenerative Medicine*.

[48] Myers F. A., Bluth M. H., Cheung W. W. (2016). Ketamine: A Cause of Urinary Tract Dysfunction. *Clinics in Laboratory Medicine*.

[49] Bureau M., Pelletier J., Rousseau A., Bernard G., Chabaud S., Bolduc S. (2015). Demonstration of the direct impact of ketamine on urothelium using a tissue engineered bladder model. *Canadian Tax Journal*.

[50] Orabi H., Saba I., Rousseau A., Bolduc S. (2017). Novel three-dimensional autologous tissue-engineered vaginal tissues using the self-assembly technique. *Translational Research*.

[51] Galbraith T., Clafshenkel W. P., Kawecki F. (2017). A Cell-Based Self-Assembly Approach for the Production of Human Osseous Tissues from Adipose-Derived Stromal/Stem Cells. *Advanced Healthcare Materials*.

[52] Grodzinsky A. J. (1983). Electromechanical and physicochemical properties of connective tissue. *Critical Reviews in Biomedical Engineering*.

[53] Wang J. H.-C., Thampatty B. P. (2006). An introductory review of cell mechanobiology. *Biomechanics and Modeling in Mechanobiology*.

[54] Buck R. C. (1980). Reorientation response of cells to repeated stretch and recoil of the substratum. *Experimental Cell Research*.

[55] Dartsch P. C., Hammerle H., Betz E. (1986). Orientation of cultured arterial smooth muscle cells growing on cyclically stretched substrates. *Cells Tissues Organs*.

[56] MacKenna D. A., Dolfi F., Vuori K., Ruoslahti E. (1998). Extracellular signal-regulated kinase and c-Jun NH2-terminal kinase activation by mechanical stretch is integrin-dependent and matrix-specific in rat cardiac fibroblasts. *The Journal of Clinical Investigation*.

[57] Gudi S. R. R., Lee A. A., Clark C. B., Frangos J. A. (1998). Equibiaxial strain and strain rate stimulate early activation of G proteins in cardiac fibroblasts. *American Journal of Physiology-Cell Physiology*.

[58] Lee A. A., Delhaas T., McCulloch A. D., Villarreal F. J. (1999). Differential responses of adult cardiac fibroblasts to in vitro biaxial strain patterns. *Journal of Molecular and Cellular Cardiology*.

[59] Adhikari A. S., Chai J., Dunn A. R. (2011). Mechanical load induces a 100-fold increase in the rate of collagen proteolysis by MMP-1. *Journal of the American Chemical Society*.

[60] Tyagi S. C., Lewis K., Pikes D. (1998). Stretch-induced membrane type matrix metalloproteinase and tissue plasminogen activator in cardiac fibroblast cells. *Journal of Cellular Physiology*.

[61] Carver W., Goldsmith E. C. (2013). Regulation of tissue fibrosis by the biomechanical environment. *BioMed Research International*.

[62] Tondreau M. Y., Laterreur V., Gauvin R. (2015). Mechanical properties of endothelialized fibroblast-derived vascular scaffolds stimulated in a bioreactor. *Acta Biomaterialia*.

[63] Fortier G. M., Gauvin R., Proulx M., Vallée M., Fradette J. (2013). Dynamic culture induces a cell type-dependent response impacting on the thickness of engineered connective tissues. *Journal of Tissue Engineering and Regenerative Medicine*.

[64] Guillemette M. D., Cui B., Roy E. (2009). Surface topography induces 3D self-orientation of cells and extracellular matrix resulting in improved tissue function. *Integrative Biology*.

[65] Chabaud S., Bolduc S. (2016). Production of a self-aligned scaffold, free of exogenous material, from dermal fibroblasts using the self-assembly technique. *Dermatology Research and Practice*.

[66] Wittmann F., Prix N., Mayr S. (2005). L-arginine improves wound healing after trauma-hemorrhage by increasing collagen synthesis. *Journal of Trauma - Injury Infection and Critical Care*.

[67] Alexakis C., Mestries P., Garcia S. (2004). Structurally different RGTAs modulate collagen-type expression by cultured aortic smooth muscle cells via different pathways involving fibroblast growth factor-2 or transforming growth factor-*β*1. *The FASEB Journal*.

[68] Wei D., Zhang L., Williams D. L., Browder W. (2002). Glucan stimulates human dermal fibroblast collagen biosynthesis through a nuclear factor-1 dependent mechanism. *Wound Repair and Regeneration*.

[69] Rioux L. E. (2010). *Département des Sciences des Aliments et Nutrinion*.

[70] Ayoub A., Pereira J. M., Rioux L.-E., Turgeon S. L., Beaulieu M., Moulin V. J. (2015). Role of seaweed laminaran from Saccharina longicruris on matrix deposition during dermal tissue-engineered production. *International Journal of Biological Macromolecules*.

[71] Lehtovaara B. C., Gu F. X. (2011). Pharmacological, structural, and drug delivery properties and applications of 1,3-*β*-glucans. *Journal of Agricultural and Food Chemistry*.

[72] Pageon H., Zucchi H., Rousset F., Monnier V. M., Asselineau D. (2014). Skin aging by glycation: Lessons from the reconstructed skin model. *Clinical Chemistry and Laboratory Medicine*.

[73] Pageon H. (2010). Reaction of glycation and human skin: The effects on the skin and its components, reconstructed skin as a model. *Pathologie Biologie*.

[74] Bjork J. W., Meier L. A., Johnson S. L., Syedain Z. H., Tranquillo R. T. (2012). Hypoxic culture and insulin yield improvements to fibrin-based engineered tissue. *Tissue Engineering Part: A*.

[75] Aijaz A., Faulknor R., Berthiaume F., Olabisi R. M. (2015). Hydrogel Microencapsulated Insulin-Secreting Cells Increase Keratinocyte Migration, Epidermal Thickness, Collagen Fiber Density, and Wound Closure in a Diabetic Mouse Model of Wound Healing. *Tissue Engineering Part: A*.

[76] Dhall S., Silva J. P., Liu Y. (2015). Release of insulin from PLGA-alginate dressing stimulates regenerative healing of burn wounds in rats. *Clinical Science*.

[77] Pallet N., Hébert M.-J. (2011). The apoptotic program promotes tissue remodeling and fibrosis. *Kidney International*.

[78] Buechler C., Krautbauer S., Eisinger K. (2015). Adipose tissue fibrosis. *World Journal of Diabetes*.

[79] Morissette A., Imbeault A., Cattan V. Strategies to reconstruct a functional urethral substitute by self-assembly method.

[80] Chabaud S., Saba I., Baratange C. (2017). Urothelial cell expansion and differentiation are improved by exposure to hypoxia. *Journal of Tissue Engineering and Regenerative Medicine*.

[81] Montesinos M. C., Desai A., Chen J.-F. (2002). Adenosine promotes wound healing and mediates angiogenesis in response to tissue injury via occupancy of A2A receptors. *The American Journal of Pathology*.

[82] Chan E. S. L., Fernandez P., Merchant A. A. (2006). Adenosine A2A receptors in diffuse dermal fibrosis: Pathogenic role in human dermal fibroblasts and in a murine model of scleroderma. *Arthritis & Rheumatology*.

[83] Chen Y., Epperson S., Makhsudova L. (2004). Functional effects of enhancing or silencing adenosine A2b receptors in cardiac fibroblasts. *American Journal of Physiology-Heart and Circulatory Physiology*.

[84] Mills G. B., Moolenaar W. H. (2003). The emerging role of lysophosphatidic acid in cancer. *Nature Reviews Cancer*.

[85] Rancoule C., Pradère J., Gonzalez J. (2011). Lysophosphatidic acid-1-receptor targeting agents for fibrosis. *Expert Opinion on Investigational Drugs*.

[86] Prodinger C. M., Reichelt J., Bauer J. W., Laimer M. (2017). Current and Future Perspectives of Stem Cell Therapy in Dermatology. *Annals of Dermatology*.

[87] Huang H., Liu J., Hao H. (2017). Preferred M2 Polarization by ASC-Based Hydrogel Accelerated Angiogenesis and Myogenesis in Volumetric Muscle Loss Rats. *Stem Cells International*.

[88] Bochaton-Piallat M.-L., Gabbiani G., Hinz B. (2016). The myofibroblast in wound healing and fibrosis: Answered and unanswered questions. *F1000Research*.

[89] Wang D., Wang A., Wu F. (2017). Sox10+ adult stem cells contribute to biomaterial encapsulation and microvascularization. *Scientific Reports*.

[90] Trottier V., Marceau-Fortier G., Germain L., Vincent C., Fradette J. (2008). IFATS collection: using human adipose-derived stem/stromal cells for the production of new skin substitutes. *Stem Cells*.

[91] Gauvin R., Ahsan T., Larouche D. (2010). A novel single-step self-assembly approach for the fabrication of tissue-engineered vascular constructs. *Tissue Engineering Part A*.

[92] Gauvin R., Guillemette M., Galbraith T. (2011). Mechanical properties of tissue-engineered vascular constructs produced using arterial or venous cells. *Tissue Engineering Part A*.

[93] Konig G., McAllister T. N., Dusserre N. (2009). Mechanical properties of completely autologous human tissue engineered blood vessels compared to human saphenous vein and mammary artery. *Biomaterials*.

[94] Carrier P., Deschambeault A., Audet C. (2009). Impact of cell source on human cornea reconstructed by tissue engineering. *Investigative Opthalmology & Visual Science*.

[95] Bouhout S., Chabaud S., Bolduc S. (2016). Organ-specific matrix self-assembled by mesenchymal cells improves the normal urothelial differentiation in vitro. *World Journal of Urology*.

[96] Taylor L. H., Latham S. M., Woolhouse M. E. J. (2001). Risk factors for human disease emergence. *Philosophical Transactions of the Royal Society B: Biological Sciences*.

[97] Chabaud S., Simard M., Gendreau I., Pouliot R., Bolduc S. (2016). Origin of Serum Affects Quality of Engineered Tissues Produced by the Self-Assembly Approach. *Scientifica*.

[98] Kaupisch A., Kennedy L., Stelmanis V. (2012). Derivation of vascular endothelial cells from human embryonic stem cells under GMP-compliant conditions: Towards clinical studies in ischaemic disease. *Journal of Cardiovascular Translational Research*.

[99] Pouyani T., Ronfard V., Scott P. G. (2009). De novo synthesis of human dermis in vitro in the absence of a three-dimensional scaffold. *In Vitro Cellular & Developmental Biology - Animal*.

[100] Deshpande M., Papp S., Schaffer L., Pouyani T. (2016). Hydrocortisone effect on hyaluronate synthesis in a self-assembled human dermal equivalent. *Journal of Tissue Engineering and Regenerative Medicine*.

[101] Pouyani T., Sadaka B. H., Papp S., Schaffer L. (2013). Triiodothyronine (T3) inhibits hyaluronate synthesis in a human dermal equivalent by downregulation of HAS2. *In Vitro Cellular & Developmental Biology - Animal*.

[102] Deshpande M., Papp S., Schaffer L., Pouyani T. (2015). Hydrocortisone and triiodothyronine regulate hyaluronate synthesis in a tissue-engineered human dermal equivalent through independent pathways. *Journal of Bioscience and Bioengineering*.

[103] Deshpande M., Papp S., Schaffer L., Pouyani T. (2014). All-trans retinoic acid is an effective inhibitor of hyaluronate synthesis in a human dermal equivalent. *Archives of Dermatological Research*.

[104] Jean J., Bernard G., Duque-Fernandez A., Auger F. A., Pouliot R. (2011). Effects of serum-free culture at the air-liquid interface in a human tissue-engineered skin substitute. *Tissue Engineering Part A*.

[105] Rochon M. H., Fradette J., Fortin V. (2010). Normal human epithelial cells regulate the size and morphology of tissue-engineered capillaries. *Tissue Engineering Part A*.

[106] Gibot L., Galbraith T., Huot J., Auger F. A. (2010). A preexisting microvascular network benefits in vivo revascularization of a microvascularized tissue-engineered skin substitute. *Tissue Engineering Part A*.

[107] Gibot L., Galbraith T., Kloos B. (2016). Cell-based approach for 3D reconstruction of lymphatic capillaries in vitro reveals distinct functions of HGF and VEGF-C in lymphangiogenesis. *Biomaterials*.

[108] Gibot L., Galbraith T., Bourland J., Rogic A., Skobe M., Auger F. A. (2017). Tissue-engineered 3D human lymphatic microvascular network for in vitro studies of lymphangiogenesis. *Nature Protocols*.

